# It's time! Ten reasons to start replicating simulation studies

**DOI:** 10.3389/fepid.2022.973470

**Published:** 2022-09-14

**Authors:** Anna Lohmann, Oscar L. O. Astivia, Tim P. Morris, Rolf H. H. Groenwold

**Affiliations:** ^1^Department of Clinical Epidemiology, Leiden University Medical Center, Leiden, Netherlands; ^2^College of Education, University of Washington, Seattle, WA, United States; ^3^MRC Clinical Trials Unit at UCL, Institute of Clinical Trials and Methodology, University College London, London, United Kingdom; ^4^Department of Biomedical Data Sciences, Leiden University Medical Center, Leiden, Netherlands

**Keywords:** replication, data analysis, research statistics, simulation study, reproduction

## Abstract

The quantitative analysis of research data is a core element of empirical research. The performance of statistical methods that are used for analyzing empirical data can be evaluated and compared using computer simulations. A single simulation study can influence the analyses of thousands of empirical studies to follow. With great power comes great responsibility. Here, we argue that this responsibility includes replication of simulation studies to ensure a sound foundation for data analytical decisions. Furthermore, being designed, run, and reported by humans, simulation studies face challenges similar to other experimental empirical research and hence should not be exempt from replication attempts. We highlight that the potential replicability of simulation studies is an opportunity quantitative methodology as a field should pay more attention to.

Statistical simulation studies are computer experiments in which data are generated using computer algorithms (1, 2). They are an important tool for investigating the properties of data analysis methods and often inform choices about how empirical data will be analyzed (3). Replicability has been a prominent issue in the social and biomedical sciences over the past decade, yet simulation studies have, until recently, been largely exempt from replication attempts. Perhaps this is because the researcher who is performing a statistical simulation study can set every parameter at will and knows the data generating mechanism (or algorithm) that was used. The apparent lack of human manipulation in simulation studies lends them an air of infallibility and some, therefore, regard simulation studies as equivalent to mathematical derivations, carved in stone (4).

We argue, however, that simulation studies have more in common with empirical research than might meet the eye. The replication of a published simulation study, i.e., writing and running new code based on the description provided in the original publication (5), might produce incompatible results for many of the same reasons seen in empirical research in the social and biomedical sciences. These reasons include human error, design choices, as well as suboptimal execution and reporting of simulation studies, which may jeopardize their theoretically perfect replicability. Figure 1 and Box 1 summarize 10 issues regarding replicability that we have identified. In this article, we discuss each of these issues in more detail. With this, we hope to spark a discussion on the need for replication of statistical simulation studies.

**Figure 1:**
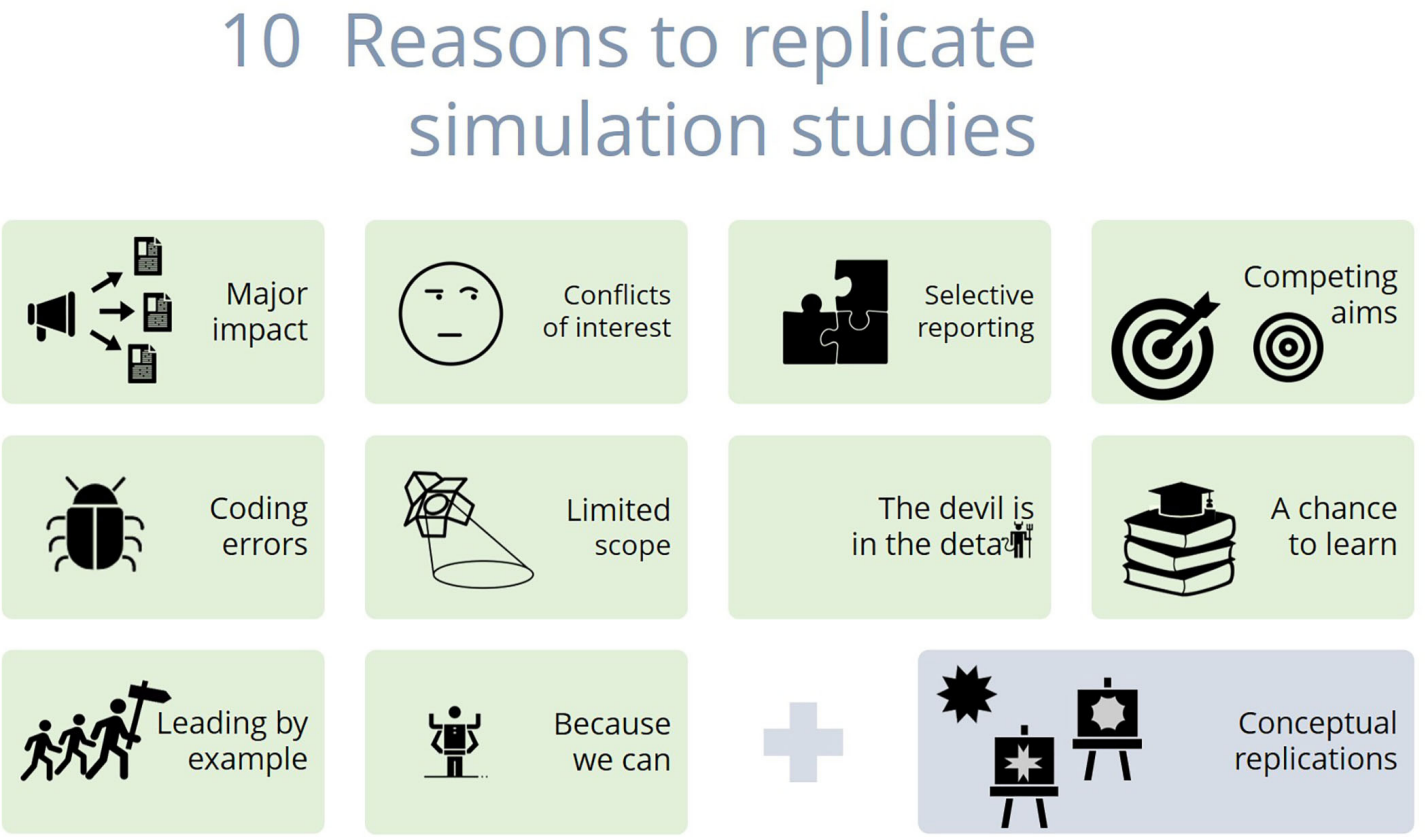
Ten reasons to replicate simulation studies. Image description: The figure shows 11 boxes. Each box contains one of the reasons to replicate simulation studies together with an icon visualizing the corresponding reason. (1) Major impact: A megaphone with arrows pointing to articles; (2) Conflict of interest: A face critically glancing to the side; (3) Selective reporting: A puzzle with a missing piece; (4) Competing aims: Two bulls' eyes, one of them with an arrow in the center; (5) Coding errors: A bug; (6) Limited scope: A spotlight; (7) The devil is in the detail: The last two letters of “detail” are replaced with a tiny devil; (7) A chance to learn: A pile of books with a graduation cap on top; (8) Leading by example: three stick figures marching behind each other, the first one carrying a road sign; (10) Because we can: A figure with four arms; (11) (Bonus reason) Conceptual replications: A star and two easels, each easel carrying a picture of a different star.

**Box 1:** 10 Reasons to replicate simulation studies.

**Reasons to replicate simulation studies**	**Explanation**
1) Major impact	Highly cited simulation studies can influence many subsequent studies and form the foundation of data analysis across different research fields.
2) Conflicts of interest	Researchers conducting simulation studies may be invested in certain methods which may bias design choices and how result are presented.
3) Selective reporting of results	Journal restriction may limit the amount the result being presented, yet favoritism toward one method can bias focus of reported results.
4) Competing aims	Results of simulation studies are relevant for different audiences: while methodologically oriented readers may be interested in general properties of investigated methods, applied researchers may look for guidance for their particular use case.
5) Coding errors	Although coding errors can happen to anyone, there is generally still a lack of code review and often unavailability of simulation code that allows for (external) checking code.
6) Limited scope	Since the number of simulation scenarios is finite, generalizing to a particular research setting be beyond the scope of the simulation study, thus requiring replication for these further scenarios.
7) Importance of details	Reported information may be insufficient for comprehensive assessment of (results of) simulation studies - even for dedicated peer-reviewers.
8) Insights as individuals and as a field	Replication encourages reflection on reporting standards and practices such as making code publicly available, code review, and pre-registration of simulation studies.
9) Lead by example	Methodologists have the chance to practice what they preach.
10) Because we can	There are no financial, logistic, historical, or ethical constraints to replication (although there are often serious time and funding constraints to individuals that produce them!)

##  1) Simulation studies can have a major impact

Methodological decisions for the choice of a certain method, or the use of a specific cut-off or rule of thumb can quickly become mainstream, particularly if the proposed method or rule is convenient. A simulation study that inspired a given recommendation often reaches seminal status and thousands of citations. Famous examples are Hu and Bentler (6) whose simulation study of cut-off values for fit indices in structural equation models was cited over 70,000 times or Peduzzi et al. (7) whose simulation study promoting the 1 in 10 rule for the events-per-variable in logistic regression has accumulated over 6,900 citations as of February 2022. After a data analytical recommendation has reached the state of common practice, these citation counts are likely an underestimation (8). The impact of simulation research investigating the performance of statistical methods furthermore crosses discipline borders. Whenever the method under investigation is of broad application in various fields, so might be a simulation study investigating its properties.

##  2) Authors of simulation studies are not immune to conflicts of interest

Most researchers are genuinely interested in the progress of science and happy to understand the limitations of their theories, hypotheses, or methods in order to refine and evolve them [for a commendable example see (9)]. Yet the feeling of ownership and pride in the context of one's own scientific contributions make us prone to engaging in questionable research practices like HARKING, cherry picking, or p-hacking [see, e.g., (10) for an explanation of these terms]. We have no reason to believe that quantitative methodologists are an exception, as they may be biased toward their own methods just as empirical researchers are biased toward their theories. Even the slightest perceived threat of reputation or citation loss might make authors of simulation studies conflicted when deciding about, for example, which methods to compare, which scenarios to study, which performance measures to compare the methods on, and how to present and interpret the results (3). Confirmation bias makes it furthermore less likely for errors to be detected whenever the simulation code yielded a “favorable” result.

In recent years, the *Replication/Reproducibility crisis* has made evident that pressures outside of the process of scientific discovery can play an overdue role in the design, reporting, and publication of results. When the primary directive of academics is to publish diligently and copiously, it should not come as a surprise if lapses in quality and attention, whether intentional or not, become common over time and the conclusions of published papers become suspect. For the case of simulation research, this could take any number of forms, such as highlighting the aspects of a simulation study where one's preferred method outperforms others, or selectively choosing or reporting specific simulation conditions aimed at guiding the conclusions intended by the researchers. *Salami slicing*, the practice of obtaining multiple publications from a single dataset (11), is another aspect where simulation research could follow the current academic incentive structure. A simulation study could easily be broken into smaller parts or “mini-simulations” with the aim of maximizing the number of publications that can be produced from a single simulation study.

##  3) Selective reporting is the norm rather than the exception in simulation studies

Simulation studies frequently compare multiple statistical methods on multiple performance measures. Furthermore, full-factorial designs, i.e., the crossing of all experimental conditions, are often regarded as the gold standard. The potential number of scenarios that might be included in any given simulation study is ever-increasing due to the evolution of computing capacities of modern computers and the growing availability of high performance clusters and cloud computing. As a result, authors of simulation studies are faced with the challenging task of taming an overwhelming number of results. Elaborate accounts are limited by the restrictions of scientific journals and author guidelines on the number of figures and/or tables. Although online supplements provide the opportunity to comprehensively present many results, these are less frequently subjected to peer-review, often formatted by the authors, and sometimes hard to access. Other may fall prey to temptations of a clean story and aesthetic standards (12) and omit “less informative” configurations of the data-generating mechanism (2). It is arguably fair that authors wish to focus on when methods perform particularly poorly or well, but unless readers are fully aware of all the data-generating scenarios actually explored and the entailing adequacy of methods for unreported configurations, this may give a false impression.

##  4) Simulation studies serve two masters

In addition to the overwhelming quantity of data that a simulation study author has to make digestible for readers, the readership they have to serve can be divided into two broad groups—albeit caricatured—with different needs and preferences. On the one hand, simulation studies are typically written and reviewed by methodologists, and thus the writing will suit an audience of fellow methodologists, who are interested in the broad picture. They would like to learn about general properties of a given statistical method: when it performs well, when it performs poorly, and why. On the other hand, simulation studies provide guidance for applied researchers, who might be interested in a more specific use case, i.e., a particular combination of simulation factors. They would like to know which of the compared methods is most suitable for their particular research data, for example in terms of design, sample size, and expected structure of the data. A method that works well for a randomized trial but poorly in non-randomized settings will not concern the applied researcher if they are analyzing a randomized trial (13). Providing a broad narrative about a simulation study's results in addition to results for specific scenarios requires care in the analysis, presentation, and interpretation of results, akin to challenges faced by other empirical researchers. A replicator's perspective may be mainly one or the other leading to different choices in data analysis, presentation, and (in particular) interpretation, potentially altering some conclusions or providing different insights.

##  5) Simulation studies might be run by computers but the code is written by humans

Asking for replicability of simulation studies might seem absurd. After all they are merely the result of computer code that can be rerun at will. There are three problems with this notion. First, rerunning computer code requires the availability of computer code. However, computer code is almost never publicly available (2, 14). Second, the computer code has to be error free. Mistakes can happen in any human endeavor, with computer code being no exception. Third, the computer code has to match the intended simulation design. Methodological intentions have to be correctly translated into computer code. Code including a design-implementation-gap is reproducible in the sense that rerunning the code would produce the same results. However, it is not replicable. A replicator trying to accurately translate the intended design into code would likely get diverging results. Such a gap can be of a more systemic nature and affect multiple simulation studies implementing similar concepts [see (15) for an example] or can closely resemble coding errors. The latter was the case in the study by Schönbrodt and Perugini (16) who published a corrigendum for their simulation study investigating the sample size at which correlations stabilize. It had been brought to the researchers' attention that the description provided in the paper did not match the accompanying code. While fortunately not altering the main conclusions the authors took the corrigendum as an opportunity to highlight the necessity of open reproducible code.

A different source of coding errors might be the software implementation of a given method (e.g., in a package used in the simulation code). This may lead to a given method appearing to perform poorly, while, in fact, the method is fine and merely the specific software implementation faulty. Naturally, authors will take more care to check the implementation of their own method than competing methods.

##  6) We cannot simulate every possible scenario

In statistical simulation studies, synthetic data sets are usually generated according to one or more specific conditions (often termed scenarios). Different data analysis strategies are then applied to each of the resulting data sets. For promising methods, generalizability of results is a primary concern, since the parameter space that can be covered by a single simulation study is, by definition, finite. Conclusions about the performance of a given method therefore hold as long as those specific conditions under which it was studied are met and do not necessarily extend to other conditions or settings. For the applied researcher faced with a data set (see point 4), it would be very unusual to know which specific scenarios are most relevant to the study at hand. As obvious as this may seem, it is hard to say how broadly the results of a simulation study may apply. While it is possible to model the results of simulation studies, e.g., using a meta-model (17), this typically involves extrapolation. Instead of speculating about the generalizability of the results of simulation studies, replication for these further scenarios provides a means of direct investigation.

##  7) The devil is in the detail

Peer-review is an important element in ensuring the quality and integrity of research. One aspect that is consistently overestimated is a peer-reviewer's ability to detect errors or hidden assumptions. Verification reports and red teams provide an insight into the effort that is required to spot inconsistencies and question decisions that might seem self-evident when presented in the coherent story line of a published manuscript (18, 19). Only when getting our hands dirty, diving deep into the details, and actually retracing each step *via* replication can less obvious threats to validity be uncovered. An example for such an influential detail is the simulation study by Van Smeden et al. (20) investigating the one variable per 10 events criterion for binary logistic regression. The authors showed that simulation results were highly dependent on the existence of artificial data sets where the outcome can be perfectly predicted by the covariates (*separation*).

Differences in the replicators' perspective and the potential changes in simulation results echo the issue of *hidden moderators* that has been brought forward within the Replication Crisis movement as an attempt to explain why empirical studies do not replicate (21). As one of several explanations, the *hidden moderators* hypothesis argues that study features which may not have been consciously accounted for in the original design play an important role in obtaining similar results in empirical experiments. Without them, the designs are not directly comparable and differences in the results should be expected. If a replicator has a different perspective on the design of a simulation study, such as conducting a conceptual replication to see if the same results generalize, it may seem intuitively appealing that the results should change (i.e., different designs, different results). Nevertheless, it is important to highlight the fact that if unplanned idiosyncrasies of the design are crucial for the results to be observed, then said results are probably less robust and applicable to real life settings than originally thought.

##  8) Replication is a chance to reflect both as individuals and as a field

The replication crisis has brought about a substantive change to how science is conducted. It initiated reflections on common practices such as reporting guidelines, the role of peer-review and publication formats. Reporting guidelines have for decades been lamenting the poor design and reporting practices of simulation based research (1, 2, 22–24). Embracing replication and the subsequent change of perspective, from telling your own story to understanding and recreating others' research, might facilitate the long-overdue adoption of some best practices such as open code, code review, and pre-registration. In addition to recognizing the merit of the best-practice recommendations that emerged from the replication crisis in psychology and learning how they might serve us in the context of simulation studies, we might learn our very own lessons and derive reforms specific to simulation studies.

##  9) Research for research has the chance to lead by example

The study of research methodology is inherently prescriptive. Already in 1975, Hoaglin and Andrews (22) pointed out the hypocrisy of methodologists not adhering to their own advice when it comes to simulation studies. The very people who often provide statistical advice to applied researchers and lament the brevity of method sections or lack of rigorous reporting fall prey to the same trappings. It would certainly be nice if simulation studies contained not only guidance for data analysis and study design but were additionally exemplary in transparency and data and code sharing (25).

##  10) Because we can

Large swathes of empirical research are inherently unreplicable. Financial, logistical, historical, or ethical reasons can prohibit the direct replication of published research. None of these constraints apply to simulation studies. Generating artificial participants does not require ethical review board approval; we do not need to concern ourselves with participant burden, the costs of large trials, or the inability to recreate the effects of unique historical events. Neither do we face struggles to recruit participants from a small population or have to wait a decade for the long-term effects of interventions to manifest. We are free to openly share our code and artificial data without any concerns for privacy. While exact replication is never possible in empirical research, it is possible in simulation studies due to knowledge of all components involved. This allows examination of the root of any discrepancies that might arise in replication attempts. We can improve the research quality of an entire field from the comfort of our desks at the minimal cost of computational time and power. Given the lack of obstacles and costs, combined with the potential benefit, we shouldn't think twice about replicating simulation based research.

## Discussion

We have argued that simulation studies are subject to the same human error, researcher degrees of freedom, conflicts of interest, questionable research practices, human bias, and fallibility as any other empirical research project. Their analysis and interpretation is neither neutral nor self-evidently correct. Therefore, just like empirical research, simulation studies warrant replication.

We acknowledge that increasing efforts to replicate simulation studies would take time away from other research activities. This obviously holds both for an individual researcher and the entire (academic) research community. However, particularly those simulations that have a substantial impact within a research community (e.g., based on number of citations) should be replicated, because, if they cannot, this would potentially impact many conclusions of other studies too. As a research community it would therefore be worthwhile to invest in replication of those influential simulation studies.

Unfortunately, meta-research in the area of simulation studies is rare. We hope that our paper will raise awareness of this issue and inspire research in that direction. Research challenging individual simulation studies often addresses the data generating mechanism [see (26) for an example]. Extensions often address modifications of methods which are compared. More nuanced reflections of the implementation likely go unnoticed due to the lack of technical documentation/computer code.

Although further evidence needs to be collected to understand the scope of this issue, investigating selective reporting in computational studies is difficult. Hutson (27), for instance, has commented on how the Artificial Intelligence (AI) community is grappling with its own issues of replicability, with emphasis on how time-consuming and effort-intensive it is to reproduce and understand the conditions in which the algorithms under scrutiny operate. Without access to the source code or training datasets, AI researchers interested in further testing algorithms beyond the conditions published are left with no choice but to reproduce, from scratch, every single computer study they wish to test. Boulesteix et al. (28) offer a type of indirect evidence regarding the problem of selective reporting. In their systematic review of 59 computational articles in the field of supervised learning, all articles introducing new methodologies (43 of them) had no negative conclusions and only presented cases or simulation conditions where the new methodology outperformed existing ones. The remaining articles centered on comparing existing methodologies and, in those articles, no algorithm outperformed the other ones in all conditions. There is a very evident mismatch between the choice of conditions when the authors are invested in the methodology vs. when they are not, which may suggest selective reporting is at hand.

While we predominantly used the term *replication* in the present article, we also see the need for *repetition* and *reproduction* (5). *Repetition* by the same team using the same setup ensures that the original simulation pipeline is robust. Repetition can reduce or prevent slip-ups, such as accidentally running an old version of the code or including the wrong version of a figure in the manuscript. *Reproduction* involves a different team running the original code, and is a first step toward code-review. This is already common practice at some research institutes and is increasingly finding its way into the peer review process (e.g., at Behavior Research Methods, Biometrical Journal, Meta-Psychology to name but a few). An example in computer science can be found at http://repeatability.cs.arizona.edu/. These commendable examples increase the reputation of code as a research product. *Replication* as defined above can be done for the existing body of research, for which code might not be available - or no longer functional due to evolving software environments. This approach is implemented in the RepliSims project (https://replisims.org): an international interdisciplinary collaboration of quantitative methodologists that aims to replicate influential simulation studies from the biomedical and social sciences and to learn from that endeavor. Nosek and Errington use a broader understanding of *replication* which they define as anything that changes or confirms our conclusion of the research question (29). Simulation studies are regularly interpreted as answers to very broad research questions even though they just cover a very specific interpretation of the problem. Common examples might be the simulation of publication bias or multivariate non-normality (26) the operationalization of which are highly dependent on (subjective) assumptions of the original author. We argue that the replication of simulation studies should therefore also be extended to *conceptual replications* where the principles that inspired a data generating mechanism might be implemented in alternative ways.

**Figure 2:**
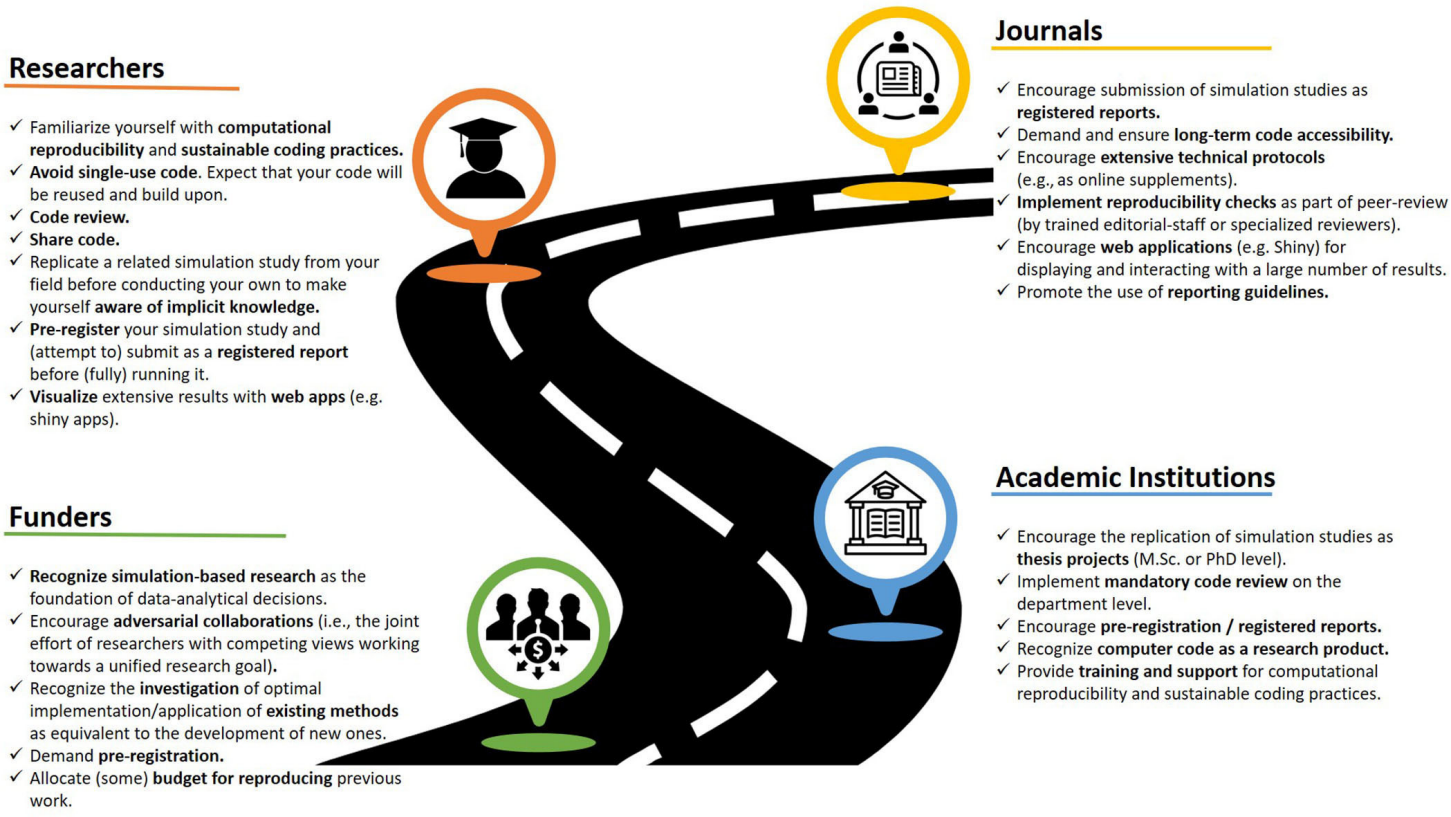
A roadmap for improving (the replicability of) simulation based research.

The resources listed in Box 2 should be considered as a starting point for replicating simulation studies. In addition, in the process of increasing the reproducibility of simulation based research, we might find that adopting modern publishing formats such as registered reports, verification reports (18) or interactive visualization tools (30) are the next steps. Once these practices become the norm we can rise to the ensuing replication related challenges such as independent implementations, long-term reproducibility, and implementation using open source tools, as well as cumulative research by extension of the existing code base. As a research paradigm, reproducibility has experienced a number of obstacles for its widespread adoption, which is mainly the lack of access to proper funding to conduct this kind of research (31). The reproducibility of computer simulations not only contends with this, but also with the perception that this research is affordable (hence, in no need of funding) and would only yield useful insights in situations where specific methods or conclusions are valid irrespective of the context in which said methods are applied (25). The research field requires a change that does not only include individual researchers, but also their institutional environment as well as editors, peer-reviewers, funders, and publishers (see Figure 2 for a roadmap of ideas involving different stakeholders).

Box 2 **Resources for replicating simulation-based research.**

**General literature on (computational) reproducibility**

Gray CT, Marwick B. Truth, proof, and reproducibility: there's no counter-attack for the codeless. *ArXiv:1907.05947*. (2019)

Marwick B, Boettiger C, Mullen L. Packaging data analytical work reproducibly using R (and friends). *Am Stat*. (2018) 72:80–8. doi: 10.1080/00031305.2017.1375986

The Turing Way Community, Arnold B, Bowler L, Gibson S, Herterich P, Higman R, et al. *The Turing Way: A Handbook for Reproducible Data Science (Version v0.0.4)*. Zenodo (2019). doi: 10.5281/zenodo.3233986

**Verification reports**

Chambers CD. Verification reports: a new article type at Cortex. *Cortex*. (2020) 2020:S0010945220301738. doi: 10.1016/j.cortex.2020.04.020

**Template for a replication report of a simulation study**

The Replisims project: https://Replisims.org/ **RedTeams**

Lakens D. Pandemic researchers - recruit your own best critics. *Nature*. (2020) 581:121. doi: 10.1038/d41586-020-01392-8

**Reproducibility checks**

Code check: https://codecheck.org.uk/

**Tips on reporting simulation studies**

Morris TP, White IR, Crowther MJ. Using simulation studies to evaluate statistical methods. *Stat Med*. (2019) 38:2074–102. doi: 10.1002/sim.8086

Burton A, Altman DG, Royston P, Holder RL. The design of simulation studies in medical statistics. *Stat Med*. (2006) 25:4279–92. doi: 10.1002/sim.2673

Smith MK, Marshall A. Importance of protocols for simulation studies in clinical drug development. *Stat Methods Med Res*. (2011) 20:613–22. doi: 10.1177/0962280210378949

**Visualization of simulation results**

Gasparini A, Morris TP, Crowther MJ. INTEREST: INteractive Tool for Exploring REsults from Simulation sTudies. *arxiv.org/abs/1909.03813* (2020). doi: 10.48550/arXiv.1909.03813

## Conclusion

It is time to embrace the methodological knowledge we have as a field and adhere to our own advice. For methodologists, the promotion and adherence to principles that ensure credibility, validity, and generalizability of the results of scientific research should be self-evident: clear and extensive description of methodology in the form of a technical simulation protocol, public availability of computer code, and mandatory code review are the very first steps toward a firm foundation. We hope to stimulate methodologists to seriously consider the replication of simulation studies and provide a basis for fruitful discussion among all stakeholders. After all, simulation studies that are worth publishing are worth replicating.

## Data availability statement

The original contributions presented in the study are included in the article/supplementary material, further inquiries can be directed to the corresponding author/s.

## Author contributions

The authors assumed the following roles according to the Contributor Roles Taxonomy (credit.niso.org): AL: conceptualization, visualization, and writing—original draft, review and editing. RG: conceptualization, supervision, and writing—review and editing. TM: investigation, visualization, and writing—review and editing. OA: investigation and writing—review and editing. All authors approved the final submitted version of the manuscript.

## Funding

AL was funded by a personal grant from the German Academic Scholarship Foundation. TM was supported by the UK Medical Research Council (grant numbers MC_UU_12023/21 and MC_UU_12023/29). RG was supported by the Netherlands Organization for Scientific Research (ZonMW-Vidi project 917.16.430) and the LUMC.

## Conflict of interest

Author TM declares that he consults for Kite Pharma, Inc.

The remaining authors declare that the research was conducted in the absence of any commercial or financial relationships that could be construed as a potential conflict of interest.

The handling editor AS declared a shared affiliation with the author TM at the time of review.

## Publisher's note

All claims expressed in this article are solely those of the authors and do not necessarily represent those of their affiliated organizations, or those of the publisher, the editors and the reviewers. Any product that may be evaluated in this article, or claim that may be made by its manufacturer, is not guaranteed or endorsed by the publisher.
